# Optimization of Moving Cone Liner Dynamics and Health Status Prediction for Cone Crushers

**DOI:** 10.3390/s26113449

**Published:** 2026-05-29

**Authors:** Minghao Li, Ruixin Fu, Dongsheng Wu, Lijuan Zhao

**Affiliations:** 1 School of Mechanical Engineering, Shenyang Ligong University, Shenyang 110159, China; 2 Science and Technology Development Corporation, Shenyang Ligong University, Shenyang 110159, China; 3 College of Mechanical Engineering, Liaoning Technical University, Fuxin 123000, China; 4 Liaoning Provincial Key Laboratory of Large-Scale Mining Equipment, Fuxin 123000, China

**Keywords:** cone crusher, mantle liner, butterfly optimization algorithm, parameter optimization, health state prediction

## Abstract

As a core crushing equipment in mining, building materials, and related industries, the cone crusher relies heavily on the optimal design and health state prediction of its mantle liner to enhance equipment reliability and reduce maintenance costs. This paper proposes a comprehensive approach integrating dynamic modeling, intelligent optimization, and health prognosis. First, a virtual prototype model is established based on laminated crushing theory and multibody dynamics simulation to analyze the motion and force characteristics of the mantle liner. Second, for the two key parameters—counterweight mass and motor speed—an improved butterfly optimization algorithm (IBOA) incorporating Cauchy mutation and an adaptive weight is proposed to achieve efficient global optimization. Furthermore, vibration signal features are extracted at different wear stages; a comprehensive health indicator curve is constructed by combining PCA dimensionality reduction with adaptive feature fusion (ASFF), and the Weibull degradation model is employed for life extrapolation prediction. Finally, fuzzy C-means (FCM) clustering is applied to autonomously partition the health states. Parameter optimization reduces the standard deviation of the force acting on the mantle liner by approximately 15.4%, markedly improving system operational stability. Health prognosis reveals that the liner enters a faulty state after 785 h, and the health condition is effectively classified into four stages: healthy, good, degraded, and faulty. The results demonstrate that the proposed optimization and health prognosis methods can effectively improve the operational efficiency and reliability of cone crushers, exhibit favorable engineering applicability, and provide a quantitative basis for condition monitoring and maintenance decision-making.

## 1. Introduction

The cone crusher is a key piece of equipment in secondary and tertiary crushing processes, widely used in mining, metallurgy, and building materials. As a core wear part, the mantle liner directly withstands crushing and impact loads from the material; its performance degradation significantly affects crushing efficiency, energy consumption, and overall equipment reliability [1]. According to statistics, crushing and grinding operations account for approximately 4–5% of global electricity consumption, yet the energy utilization efficiency in these processes is often below 3%. Therefore, improving the performance and efficiency of crushing equipment is of great significance for achieving green and intelligent manufacturing.

In recent years, considerable research has been devoted to the structural optimization of cone crushers. Yamashita et al. [2] presented a systematic review of modeling methods and control strategies for cone crushers in the mineral processing and quarrying industries, outlining a research framework based on DEM, multibody dynamics, and various control algorithms. Regarding structural optimization, early studies primarily focused on improving the chamber profile and liner geometry to increase the reduction ratio and reduce energy consumption [3,4]. With advances in computer technology, the discrete element method (DEM) and the finite element method (FEM) have been widely adopted to simulate the crushing process and analyze the forces acting on components. For instance, Johansson et al. [5] combined DEM simulations with laboratory experiments to systematically evaluate cone crusher performance; Guo et al. [6] employed DEM and response surface methodology to perform multi-objective optimization of the crushing chamber, achieving significant improvements in throughput and crushing force. In the field of structural dynamics and wear, Lindqvist and Evertsson [7] developed a coupled model linking liner wear and crushing force distribution and proposed a method for predicting worn geometry, laying a theoretical foundation for studies on mantle liner degradation. Concerning operating parameter optimization, intelligent algorithms have begun to be introduced. For example, Yu et al. [8] applied a genetic algorithm–support vector machine (GA-SVM) approach to optimize mantle structural parameters, achieving favorable results. Yan Liangkai et al. [9] adjusted key structural parameters such as eccentric throw and discharge opening size, simultaneously improving efficiency and product quality while maintaining crushing capacity. However, the aforementioned works have largely focused on static structural parameters or individual operating variables. Research on the coordinated optimization of key operating parameters that directly influence the dynamic balance, vibration characteristics, and energy consumption of the equipment—such as counterweight mass and motor speed—remains insufficient. Traditional parameter settings often rely on empirical values or recommended settings, lacking quantitative guidance based on system dynamics and global optimization theory.

In the field of intelligent optimization algorithms, the Butterfly Optimization Algorithm (BOA), an emerging swarm intelligence algorithm, has attracted attention for its minimal parameters and simple structure [10]. However, it suffers from issues, including a tendency to become trapped in local optima and limited convergence accuracy. To address these shortcomings, researchers have proposed various improvement strategies, including the introduction of chaotic maps to enhance population diversity [11], the integration of Cauchy mutations to improve global exploration capability [12], and the fusion with other algorithms (e.g., Particle Swarm Optimization) to form hybrid approaches [13]. These enhancements have significantly improved BOA’s performance in tackling complex engineering optimization problems, offering new insights into cone crusher parameter optimization.

In equipment health management, the condition assessment of key components such as the mantle liner still heavily relies on periodic manual inspections during shutdowns or simple vibration threshold alarms, making it difficult to achieve early warning of progressive degradation or predict remaining useful life. Mechanical health state prediction primarily falls into two categories: model-driven and data-driven methods. Model-driven approaches are based on physical failure mechanisms and offer strong interpretability, but they struggle to establish accurate models of complex systems [14]. Data-driven methods, which leverage sensor data and machine learning algorithms to uncover degradation patterns, have become mainstream. For instance, deep learning-based remaining useful life prediction methods have achieved good results in areas such as bearings and batteries [15,16]. Although deep learning methods perform well in health prediction, they typically rely on large-scale degradation data and suffer from weak interpretability; thus, their application in the health management of cone crusher mantle liners remains limited. Existing research has mostly focused on the diagnosis of sudden failures (e.g., cracks and fractures), while there is a relative lack of studies on quantifying the progressive wear degradation of liners over long-term operation, constructing continuous health indicators, and automatically partitioning health states based on those indicators. Moreover, how to effectively extract sensitive features strongly correlated with degradation from high-dimensional, redundant raw vibration signals and fuse them into stable, monotonic health indicators remains a technical challenge in this field.

**Figure 1:**
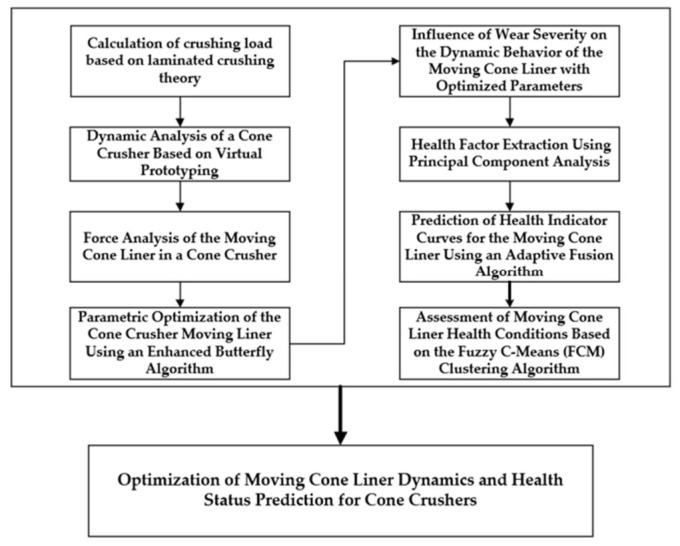
Paper technology roadmap.

In summary, existing research presents two notable gaps. First, equipment optimization has predominantly focused on static structures, while there is a lack of quantitative studies based on global optimization algorithms to explore the collaborative optimization of operational parameters (e.g., counterweight and rotational speed) that directly determine dynamic characteristics and vibration. Second, in the field of health management, there is a scarcity of integrated methods capable of extracting sensitive features from vibration signals, constructing continuous health degree curves, and achieving remaining useful life extrapolation and automatic state classification. To address these gaps, this paper aims to establish a comprehensive technical framework spanning from parameter optimization to health state assessment by integrating dynamics simulation, intelligent optimization algorithms, and data-driven prediction methods. The primary research contents are as follows: (1) Develop a high-precision virtual prototype model of a cone crusher that considers the inter-particle crushing mechanism; (2) Propose an improved butterfly optimization algorithm to realize the collaborative optimization of counterweight and rotational speed; (3) Construct a health degree assessment model based on multi-source feature fusion to achieve degradation trend prediction and automatic health state classification. The technical roadmap of this paper is illustrated in Figure 1.

The approach first analyzes the motion and force characteristics of the mantle liner through dynamic modeling and simulation. Secondly, an improved butterfly optimization algorithm is employed to co-optimize the counterweight and rotational speed, thereby reducing force fluctuations and enhancing operational stability. Finally, vibration signal features are extracted, followed by PCA dimensionality reduction and ASFF-based fusion to construct a health indicator curve. This curve is then integrated with the Weibull degradation model and FCM clustering to achieve remaining useful life prediction and state classification. These three modules are sequentially connected, together forming a complete research framework from parameter optimization to health assessment.

## 2. Dynamic Modeling and Crushing Performance Analysis of Cone Crusher

### 2.1. 3D Modeling and Virtual Prototype Development

**Figure 2:**
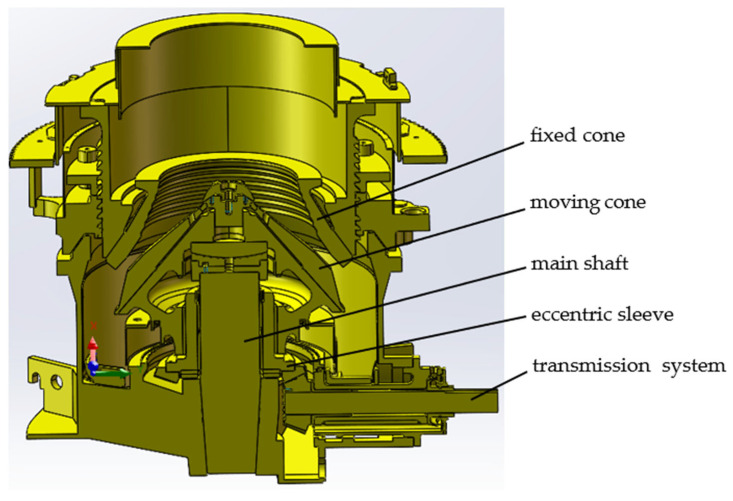
Three-dimensional model of a cone crusher.

Using Creo Parametric software, a three-dimensional model of the complete cone crusher was established based on the actual equipment drawings, as shown in Figure 2. The model includes core components such as the moving cone, fixed cone, main shaft, eccentric sleeve, and transmission system. The model is then imported into the ADAMS environment, where material properties, kinematic joint constraints, and contact force models were assigned to develop a high-fidelity virtual prototype. A piecewise function was applied to the transmission shaft drive to simulate the entire start-up, steady-state operation, and shutdown process. An eccentric counterweight was set at the bottom of the moving cone to replicate the actual unbalanced inertial forces.

When establishing a virtual prototype in ADAMS, the contact force at key contact interfaces is defined using the Impact function model.

The contact force is jointly determined by the stiffness term, damping term, and penetration depth term, with its expression given as:

(1) $$F_{n}=K{\left(\delta \right)}^{e}+C\overset{\cdot }{\delta }$$

where K represents the contact stiffness, in N/mm;

C represents the damping coefficient, in N·s/mm;

δ represents the penetration depth, in mm;

e represents the contact force exponent, which is a dimensionless parameter.

The contact friction between the drive pinion and the driven gear, as well as between the eccentric sleeve and the moving cone, is defined using the specific parameters listed in Table 1.

### 2.2. Load Calculation Based on the Theory of Inter-Particle Breakage

The crushing process of a cone crusher essentially involves the repeated compression and breakage of granular materials within the crushing chamber formed by the moving cone and the stationary cone. According to the inter-particle laminating crushing theory, the material in the crushing chamber is not crushed as individual particles. Still, it is collectively compressed into a material bed. In this process, particles interact with each other, transmitting stress and thereby achieving efficient crushing. To accurately apply crushing loads in dynamic simulations, a calculation model for crushing force was developed based on this theory [17,18].

The crushing chamber is divided into layers along the height direction. Considering factors such as material properties, particle size distribution, and compression ratio, granite (with a density of 2720 kg/m^3^ and a uniaxial compressive strength of 182 MPa) was selected as the processing material in this study. As a high-hardness hard rock, this material enables the simulation of extreme working conditions to ensure engineering safety redundancy of the conclusions. Meanwhile, it is the primary ore type processed daily by the collaborating mining enterprise, so the research findings can directly guide production. Additionally, the brittle fracture behavior of granite is highly consistent with the laminated crushing theory, ensuring the applicability of the crushing force model.

**Figure 3:**
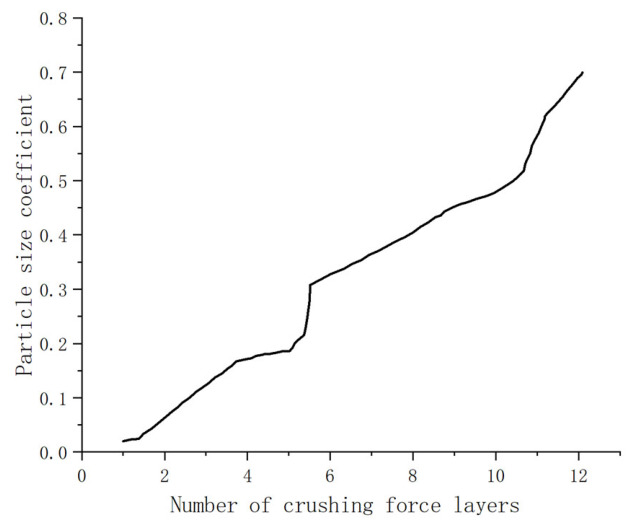
The influence of particle size coefficient on the broken layer.

The particle size distribution coefficient reflects particle uniformity, and the two are inversely related. A larger coefficient indicates greater variation in particle size, with finer particles enveloping coarser ones, resulting in insufficient force on larger particles during crushing and thus poorer crushing efficiency. Consequently, a greater crushing force is required. As illustrated in Figure 3, which shows the relationship between the particle size distribution coefficient and the number of crushing layers, a larger particle size distribution coefficient corresponds to a higher required crushing force.

The compression ratio formula is as follows:

(2) $$\varepsilon_{i}=\frac{S_{O1O2O3O4}}{S_{O1O2O5O6}}$$

where $\varepsilon_{i}$ refers to the compression ratio of the i-th layer;

$S_{O1O2O3O4}$ is the compressed area, in mm^2^;

$S_{O1O2O5O6}$ is the area before compression, in mm^2^.

In summary, the following mathematical formula for crushing force is established to express the relationship among these variables:

(3) P=f(σ,ε,l)

where P represents the amount of crushing pressure, Mpa;

σ represents the particle size distribution coefficient;

ε represents the compression ratio;

l represents the compressive strength coefficient of the material.

**Figure 4:**
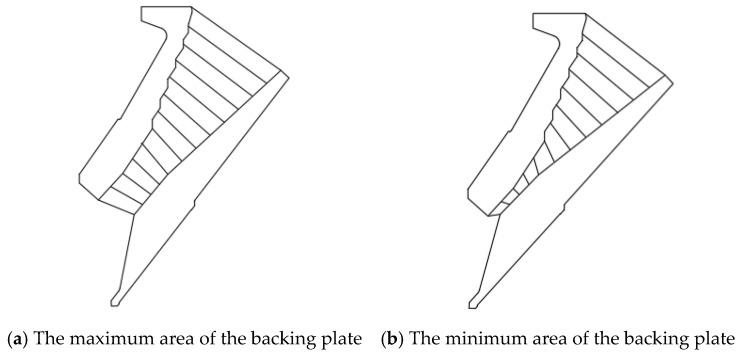
The area of the liner plate under working conditions.

**Figure 5:**
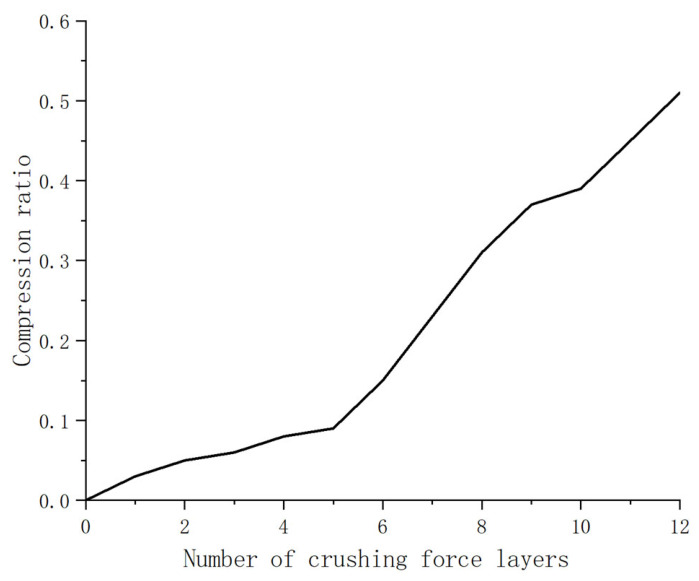
The influence of compression ratio on the fractured layer.

As illustrated in Figure 4, the ratio of the maximum to minimum area of the lining plate during the operation of the cone crusher yields the compression ratio, based on which the relationship between the compression ratio and the number of layers of crushing force is presented in Figure 5.

By establishing a calculation model for the crushing force of each layer,

(4) $$P=0.34le^{\left(6.43\varepsilon +1.9\sigma +2.04\right)}$$

In the formula, P represents the crushing force of each crushing layer, in MPa.

l represents the material compression coefficient.

ε represents the material compression ratio.

σ represents the material particle size distribution coefficient.

**Figure 6:**
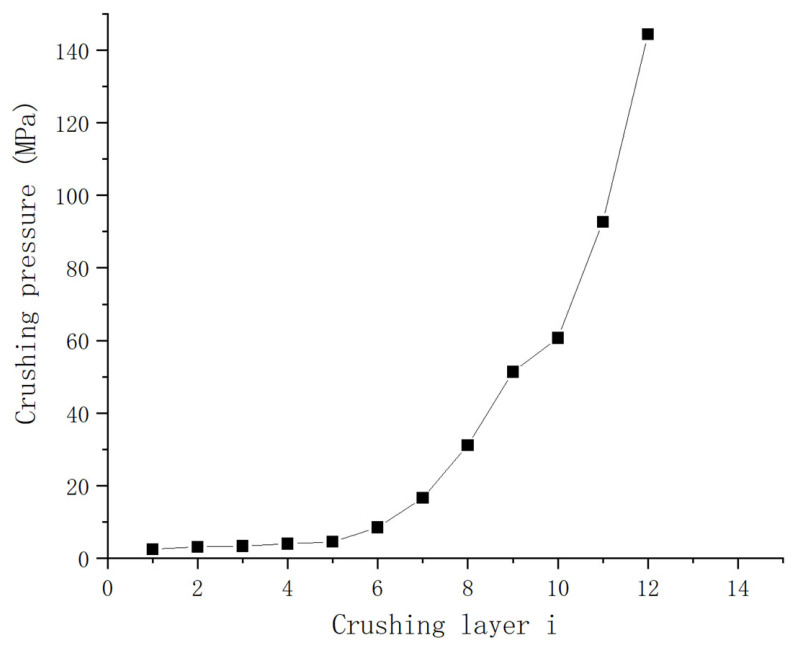
Lamination crushing force.

Taking granite as the processing object, the distribution of the compressive crushing force on the moving cone liner was obtained, as shown in Figure 6. It can be observed that the crushing force gradually increases from the feed opening to the discharge opening at the bottom, reaching its maximum near the discharge opening, which aligns with the most severely worn area of the liner in practice.

### 2.3. Dynamic Simulation and Result Analysis of Cone Crusher

**Figure 7:**
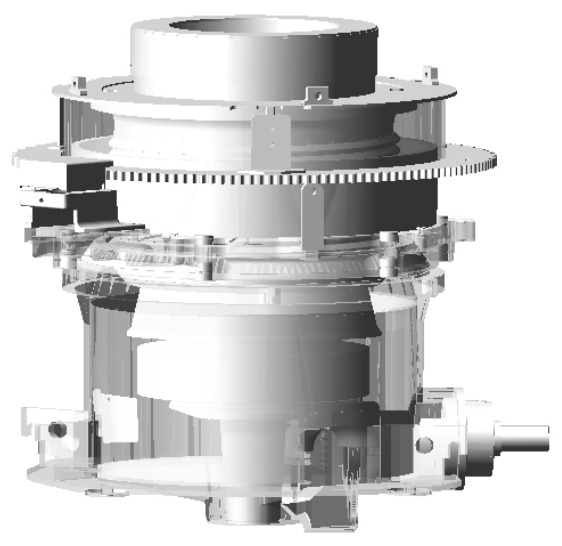
Dynamic model of cone crusher.

After completing the virtual prototype modeling and parameter configuration, the simulation duration is set to 18 s with 500 steps to carry out the dynamic simulation of the cone crusher. To accurately simulate the crushing load, the layered crushing forces calculated in Section 2.2 must be incorporated into the dynamic model. Specifically, the crushing pressure *P* of each layer is multiplied by the corresponding contact area *A* of the liner to convert it into an equivalent concentrated force *F*, which is applied as a normal load at the center-of-mass node of the moving cone liner. The completed dynamic model established in the ADAMS environment is shown in Figure 7, based on which a dynamic solution is performed to extract the motion and force characteristic data of the moving cone for analysis.

**Figure 8:**
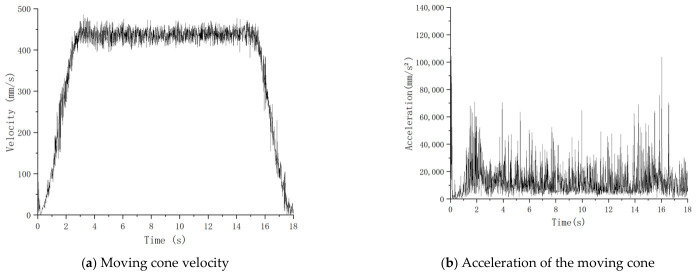
Motion characteristic diagram of the moving cone line.

**Figure 9:**
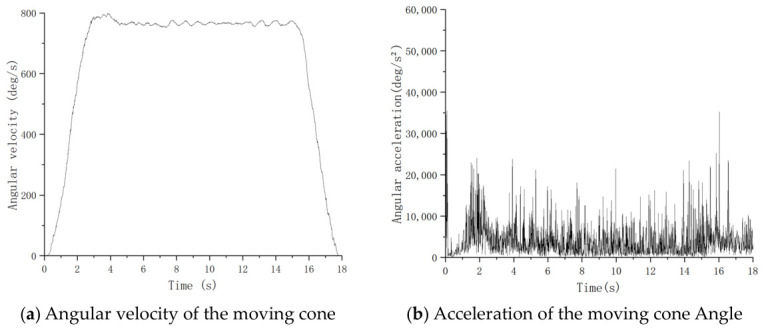
Characteristic diagram of the rotational motion of the moving cone.

In terms of the moving cone motion, Figure 8a,b show that the linear velocity of its centroid during the steady-state phase (3–15 s) has an average value of approximately 420 mm/s, with a fluctuation rate of about 3.5%, indicating smooth motion. The linear acceleration exhibits periodic fluctuations, with peaks and troughs corresponding to the compression and release phases within the crushing chamber, reflecting the periodic impact of the load. Figure 9a,b indicate that the angular velocity of the moving cone remains stable at around 780 deg/s (i.e., 130 r/min), which aligns with actual equipment operating data (120–130 r/min), thereby validating the rationality of the model setup [16]. The periodic fluctuations in angular acceleration are synchronized with those in linear acceleration, demonstrating motion coupling.

**Figure 10:**
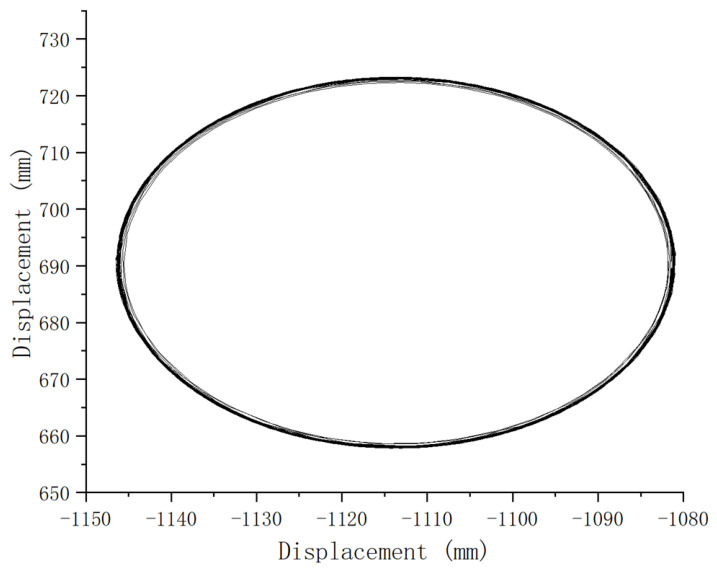
Motion trajectory diagram of the center of mass of a moving cone.

The simulation results of the moving cone centroid trajectory reveal a closed eccentric elliptical curve, as shown in Figure 10. This is a characteristic feature of the cone crusher’s moving cone motion, generated by the rotational drive of the eccentric bushing.

**Figure 11:**
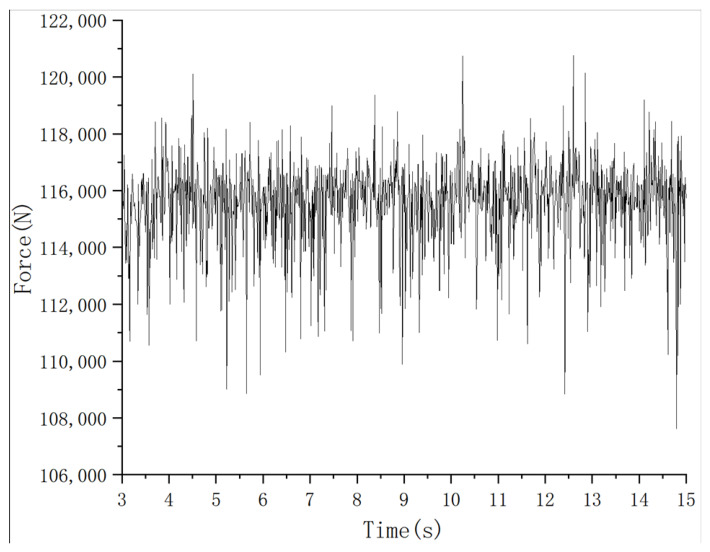
The total force curve of the center of mass of the moving cone liner under the original parameters.

Force Analysis of the Moving Cone Liner: After applying the compressive crushing force, the magnitude of the resultant force (i.e., total force) at the centroid of the moving cone liner in three directions is extracted over time. The data from the steady-state phase (3 to 15 s) are shown in Figure 11.

The force curve exhibits periodic fluctuations around a mean value of approximately 1.156 × 10^5^ N. Statistical analysis of the curve yields key indicators: a standard deviation of 1.49 × 10^3^ N, a coefficient of variation (standard deviation/mean) of 1.29%, a peak-to-peak value of 1.31 × 10^4^ N, and a crest factor (peak value/RMS value) of 1.045. These metrics indicate that under the current parameter configuration—4700 N and 350 rpm—the system operates with overall stability (as reflected by the low coefficient of variation), while still exhibiting periodic load fluctuations (peak-to-peak value accounting for approximately 11.3% of the mean) and slight impact components (crest factor slightly greater than 1). This provides a clear basis and target for further suppressing fluctuations and improving stability through subsequent optimization of counterweight and rotational speed.

## 3. Synergistic Optimization of the Moving Cone Liner Counterweight and Rotational Speed Using an Improved Butterfly Algorithm

### 3.1. Improved Butterfly Algorithm Design

The mathematical modeling of the standard Butterfly Optimization Algorithm (BOA) primarily comprises three core components: the fragrance intensity model, the individual position update model, and the search mode switching mechanism [19]. In the fragrance intensity model, the concentration of scent released by a butterfly is associated with the individual’s fitness—the higher the fitness, the stronger the fragrance and the greater the attractiveness. This relationship is expressed as

(5) $$f_{i}=c\cdot I_{i}^{a}$$

where $f_{i}$ represents the fragrance intensity of the i-th individual;

$I_{i}$ represents the fitness value of the individual;

c represents the fragrance scaling constant;

a∈[0,1] represents the sensory exponent.

Two strategies are employed for individual position updates. During global search, an individual moves toward the current global optimal position, as expressed by the following formula:

(6) $$x_{i}^{t+1}=x_{i}^{t}+r\cdot (g^{*}-x_{i}^{t})\cdot f_{i}$$

where $x_{i}^{t+1}$ represents the position vector of the i-th butterfly at the t-th iteration;

$g^{*}$ represents the position of the current global optimal individual;

r∈[0,1] represents a random coefficient;

$f_{i}$ represents the scent intensity of the individual.

Local search simulates relative motion among individuals and enhances local exploration capability through neighborhood differences. The mathematical expression for local search is

(7) $$x_{i}^{t+1}=x_{i}^{t}+r\cdot (x_{j}^{t}-x_{k}^{t})\cdot f_{i}$$

where $x_{j}^{t}$ and $x_{k}^{t}$ represent two distinct individuals randomly selected.

The search mode switching mechanism uses a random probability p to control the transition between global and local search. If rand<p, a global search is performed; otherwise, a local search is carried out, where rand∈[0,1] is a random number and p is typically set between 0.8 and 0.9. This mechanism ensures that the algorithm maintains global exploration in the early stages and progressively enhances local convergence in the later stages. By preserving search diversity throughout the iteration process, it effectively prevents the algorithm from being trapped in local optima.

Although the standard butterfly algorithm demonstrates strong global search capability when addressing complex multimodal optimization problems, it suffers from two main limitations: 1. The step size variation range of individuals remains fixed, making it difficult to adapt to the differing requirements of early-stage exploration and late-stage convergence. 2. Population diversity declines rapidly during the middle and late stages, leading to a high susceptibility to premature convergence.

To address the aforementioned issues, the Cauchy mutation and adaptive weighting mechanism are introduced to implement two key improvements [20,21], thereby enhancing the algorithm’s global search capability and dynamic convergence performance.

1. Cauchy Mutation Strategy: The Cauchy distribution is a typical long-tailed distribution capable of generating large-magnitude perturbations. Its probability density function is given as follows:

(8) $$P(x)=\frac{1}{\pi \gamma [1+{\left(\frac{x-x_{0}}{\gamma }\right)}^{2}]}$$

where $x_{0}$ represents the location parameter;

γ represents the scale parameter.

Compared with the normal distribution, the Cauchy distribution has heavier tails, which allows for longer jumps during the search process and effectively enhances the algorithm’s ability to escape local optima.

In the later stage of the algorithm, a Cauchy mutation perturbation is applied to the current optimal position $x_{i}$ to strengthen the ability to escape local optima. The update formula is as follows:

(9) $$x_{i}^{t+1}=x_{i}^{t}+\alpha \cdot C(0,1)$$

where α represents the disturbance intensity coefficient;

C(0,1) represents a random variable that follows a standard Cauchy distribution.

2. Adaptive weight strategy: The weight decays with the number of iterations to balance global exploration and local exploitation. The weight function is defined as:

(10) $$\omega (t)=\omega_{\max}-\frac{(\omega_{\max}-\omega_{\min})\cdot t}{T_{\max}}$$

where $\omega_{\max}$ and $\omega_{\min}$ represent the initial and minimum weights, respectively;

t represents the current iteration number;

$T_{\max}$ represents the maximum number of iterations.

The improved position update formula, combining the Cauchy mutation and adaptive weight is:

(11) $$x_{i}^{t+1}=x_{i}^{t}+\omega (t)\cdot r\cdot (g^{*}-x_{i}^{t})\cdot f_{i}+\alpha \cdot C(0,1)$$

**Figure 12:**
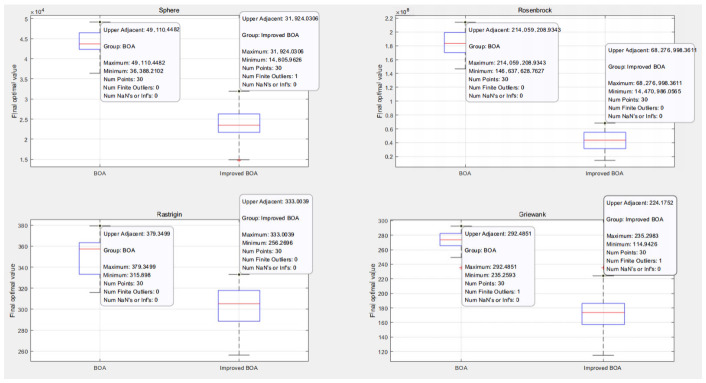
Improve the box plot of BOA and BOA test functions.

**Figure 13:**
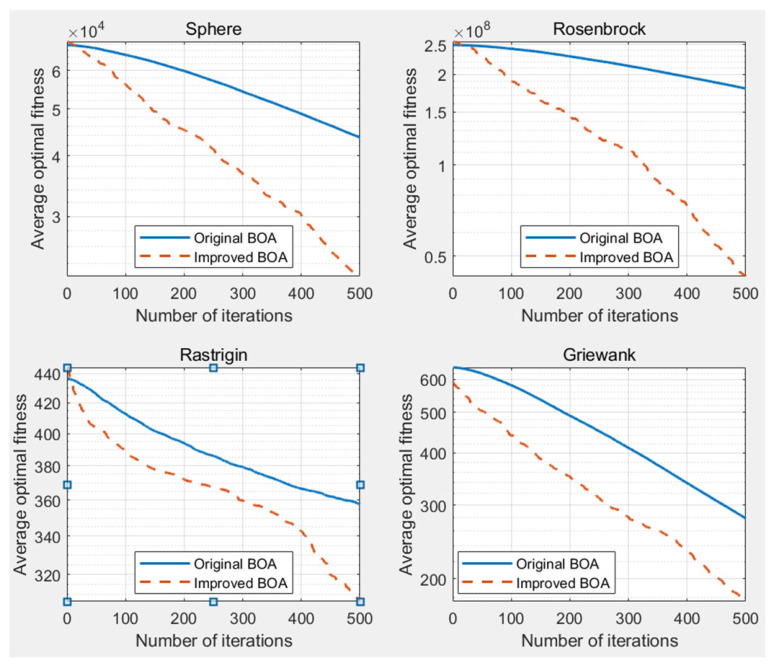
Improve the convergence curve of BOA and BOA test functions.

To verify the effectiveness of the improved butterfly optimization algorithm (Improved BOA) proposed in this study—which incorporates Cauchy mutation and an adaptive weight strategy—30 independent experiments were conducted on the original BOA and the Improved BOA using four benchmark functions: Sphere, Rosenbrock, Rastrigin, and Griewank. The final optimization results and convergence curves were compared. The corresponding boxplots and convergence curves are shown in Figure 12 and Figure 13 [20].

For the Sphere function, the median of the improved BOA decreases significantly from approximately 4.4 × 10^4^ to 2.5 × 10^4^. On the Rosenbrock function, the median fitness of the original algorithm was about 1.8 × 10^8^, while the improved BOA reduced it to 4.5 × 10^7^, corresponding to an improvement of over 70%. The advantages of the improved algorithm are also evident in multimodal functions: on the Rastrigin function, the final median value decreased from 360 with the original algorithm to 300, and on the Griewank function, it dropped from 275 to 170.

The convergence curves indicate that the improved BOA consistently achieves a faster decline across different iteration stages. For the Sphere function, at iteration t = 200, the average fitness of the original BOA is approximately 5.8 × 10^4^, whereas that of the improved BOA has dropped to 4.2 × 10^4^. In the Rosenbrock function, at t = 300, the original BOA still exhibits an average fitness of about 2.0 × 10^8^. At the same time, the improved BOA reduces to around 1.1 × 10^8^, ultimately converging to approximately 5 × 10^7^—outperforming the original algorithm’s 1.85 × 10^8^. For the Rastrigin function, at t = 200, the original BOA records an average fitness of roughly 400, compared to 365 for the improved version, which eventually converges to about 310, surpassing the original’s 360. In the Griewank function, at t = 400, the original algorithm only descends to around 330, whereas the improved BOA reaches approximately 240 and finally converges to 170, significantly outperforming the original’s 270.

Therefore, the improved Butterfly Optimization Algorithm (BOA) integrating Cauchy mutation and adaptive weights exhibits significant enhancements in global exploration, local convergence, and stability, rendering it more suitable for solving complex optimization problems. This lays a foundation for its engineering application in the collaborative parameter optimization of cone crushers. Meanwhile, regarding computational complexity: the adaptive weight calculation introduced in the improved BOA only involves scalar operations with a complexity of O(1), and the Cauchy mutation perturbation only acts on the current optimal individual with a complexity of O(D). Since neither improvement embeds additional nested loops, the single-iteration complexity of the Improved BOA (IBOA) remains O(N·D), consistent with the total time complexity O(T·N·D) of the standard BOA. This indicates that IBOA improves convergence accuracy and global exploration capability without increasing computational overhead. Relevant studies have shown that for specific engineering parameter optimization, targeted algorithm improvements can better reflect engineering applicability than comparisons based on large-scale benchmark functions [21].

### 3.2. A Collaborative Optimization Method for Counterweight and Rotational Speed Based on an Improved Butterfly Optimization Algorithm

In this study, counterweight and rotational speed are selected as optimization variables, mainly based on the following considerations: the counterweight mass directly determines the inertial centrifugal force generated by the moving cone during rotation, which is a key factor affecting the dynamic balance of the system; the motor rotational speed controls the crushing frequency and excitation characteristics. Together, these two parameters determine the force stability of the moving cone liner and are convenient for direct adjustment and control in engineering practice. Parameters such as the discharge port size and eccentricity mainly determine the cavity profile and production characteristics, which are fixed parameters determined at the design and selection stage and thus are not included in the scope of the operating parameter optimization in this work.

To perform the collaborative optimization of counterweight and rotational speed using the Improved Butterfly Optimization Algorithm (IBOA), it is necessary to first construct a continuous objective function surface that reflects the relationship between the force fluctuation characteristics of the moving cone liner and the relevant parameters. This surface is not predefined; instead, it is constructed via interpolation and fitting based on discrete dynamic simulation sample points. Considering the actual operational capacity and engineering experience of this type of cone crusher, the rotational speed range is set to 280–420 rpm, covering 80–120% of the standard operating speed (350 rpm). The counterweight range is set to 3700–5700 N, with reference to the manufacturer’s recommended value (approximately 4700 N) and a fluctuation of ±1000 N. Within these constraints, a uniform grid sampling strategy is adopted to generate multiple sets of parameter combinations, based on rotational speeds of 280, 290, 300, 310, 320, 330, 340, 350, 360, 370, 380, 390, 400, 410, and 420 rpm, and counterweights of 3700, 3800, 3900, 4000, 4100, 4200, 4300, 4400, 4500, 4600, 4700, 4800, 4900, 5000, 5100, 5200, 5300, 5400, 5500, 5600, and 5700 N, with each set corresponding to a simulation condition. Each set of parameters is input into the established ADAMS virtual prototype model. After applying the laminated crushing load, the total force curve at the centroid of the moving cone liner is extracted, and the standard deviation of the force during the steady-state phase (3–15 s) is calculated as the fitness value for evaluating the system’s operational stability. Using counterweight and rotational speed as independent variables and the force standard deviation as the dependent variable, the griddata function (with the V4 interpolation method) in MATLAB R2022a is employed to interpolate and fit the discrete sample points, thereby constructing a continuous two-dimensional response surface covering the entire parameter space. This response surface serves as the objective function surface for subsequent BOA optimization.

**Figure 14:**
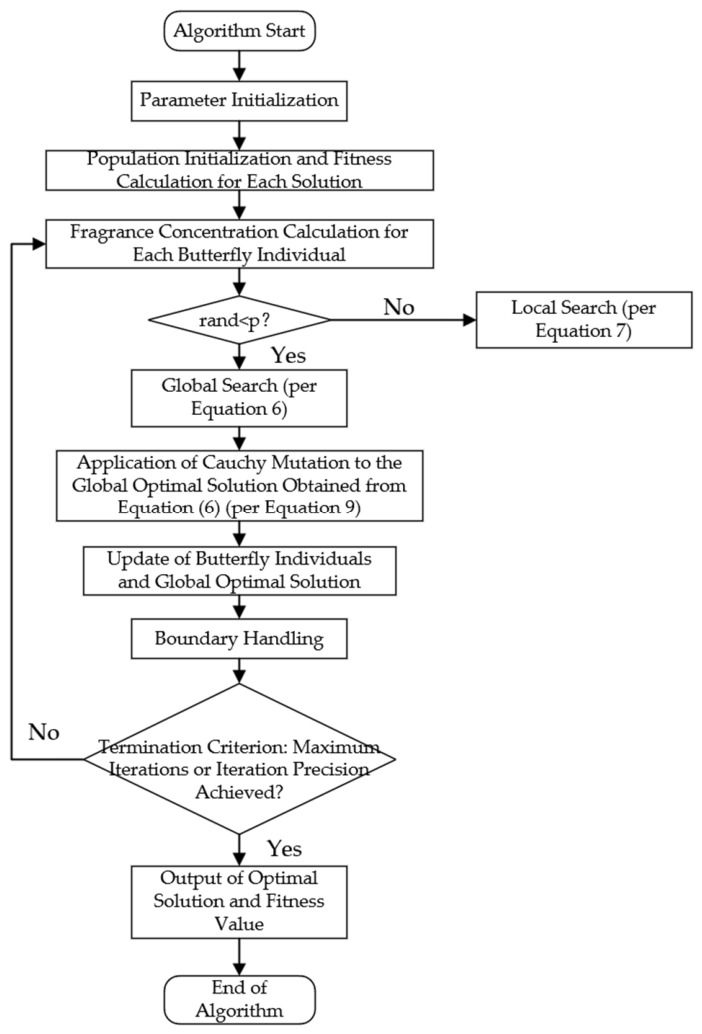
Butterfly algorithm based on Cauchy variation and adaptive weights.

Through the aforementioned approach, a limited set of dynamic simulation results is extended into a continuous and differentiable parameter-performance mapping model. This ensures global search capability within the parameter space during optimization while avoiding the repeated invocations of high-cost simulation models in each iteration, thereby significantly improving optimization efficiency. After constructing the objective function surface, the parameter co-optimization process based on the improved butterfly algorithm is illustrated in Figure 14, with the specific steps described as follows:

### 3.3. Analysis and Verification of Optimization Results for Moving Cone Liner Parameters

**Figure 15:**
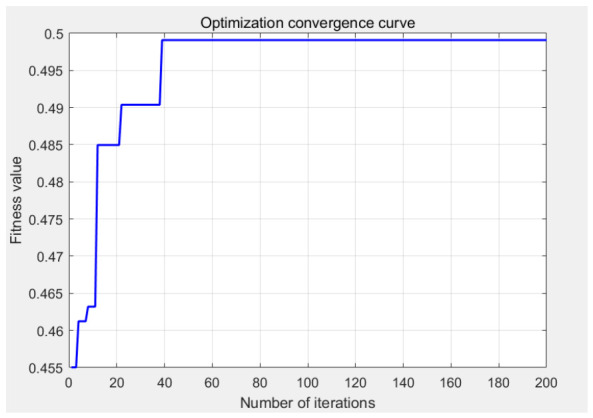
The optimized convergence curve based on the improved butterfly algorithm.

The improved Butterfly Optimization Algorithm (BOA) was employed to optimize the counterweight–rotational speed parameters, with a population size of 50 and a maximum iteration of 200. The optimization convergence curve is shown in Figure 15. The algorithm stabilizes at around 40 iterations, with the optimal fitness value corresponding to the point of minimum standard deviation.

As can be seen from the figure, the fitness value increases rapidly in the early stage of the algorithm, indicating strong global search capability and exploratory leaps. The population disperses and engages in extensive exploration via the Cauchy mutation. In the middle stage, fitness gradually stabilizes, suggesting that the population has converged to regions of high fitness and shifted to local, fine-grained search. The adaptive weight significantly reduces the step size in the search, thereby improving optimization accuracy. After approximately 40 iterations, the curve becomes stable, with the fitness value remaining around 0.5, indicating that the algorithm has converged to the global optimal solution region.

**Figure 16:**
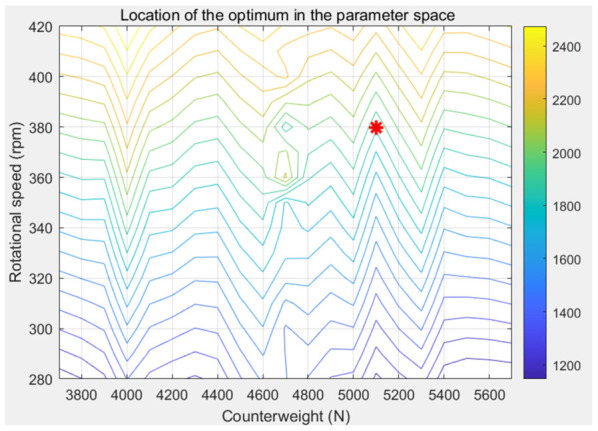
The distribution position of the optimal solution in the two-dimensional space of counterweight and rotational speed.

As illustrated in Figure 16, the optimal solutions obtained by the Cauchy mutation and adaptive weight butterfly algorithm are distributed within the two-dimensional parameter space of counterweight and rotational speed.

As illustrated in the figure, the standard deviation exhibits a distinctly nonlinear distribution across the entire parameter space, with multiple local minima forming in several regions. In the area where the counterweight ranges from 3700 to 4400 N and the rotational speed exceeds 400 rpm, the contour lines are densely distributed, and the standard deviation consistently falls within the range of 2000 to 2400. This indicates significant fluctuations in force amplitude and unstable dynamic loading in this interval. In contrast, in the region where the counterweight ranges from 4700 to 5200 N and the rotational speed ranges from 360 to 390 rpm, the standard deviation decreases to 1200–1500, indicating a low-value zone across the parameter domain. This suggests that the dynamic cone system experiences smaller force fluctuations in this region, leading to more stable operation.

The algorithm ultimately yields the optimal solution at a counterweight of 5100 N and a rotational speed of 381 rpm. This solution corresponds to the minimum standard deviation and the highest fitness, indicating that the system achieves efficient crushing while maintaining favorable dynamic balance.

**Figure 17:**
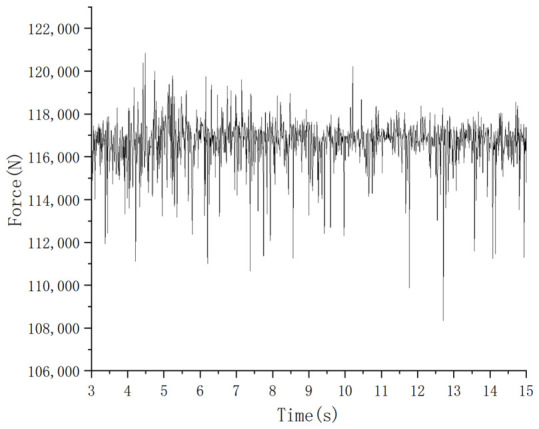
The total force curve of the center of mass of the moving cone liner under the optimal solution.

The optimal parameters—a rotational speed of 381 rpm and a counterweight of 5100 N—were entered into the virtual cone crusher model in Adams. The resulting force data acting on the center of mass of the moving cone liner from 3 to 15 s are presented in Figure 17, and a comparison of key indicators is provided in Table 2.

Analysis of the data in Table 2 reveals that after optimization, the core indicators characterizing force fluctuations—namely, the standard deviation and the coefficient of variation—decreased significantly by 15.4% and 16.3%, respectively, indicating a substantial improvement in the stability of system operation. The reduction in peak-to-peak value suggests a decrease in the amplitude of force fluctuations. The crest factor approaches unity, implying that the impact components in the signal have been attenuated, resulting in a smoother force process. The average force increased slightly (by 0.9%), which remains within a reasonable range, indicating that the optimization did not compromise crushing capacity; instead, it may have slightly enhanced energy transfer efficiency due to more stable operation. In summary, the parameter optimization based on the improved BOA effectively achieved the objective of enhancing the cone crusher’s operational stability.

## 4. Health State Prediction of the Moving Cone Liner in Cone Crushers

### 4.1. Wear Modeling of the Moving Cone Liner and Vibration Signal Acquisition

Based on the force interaction between the material and the liner, a correction coefficient is introduced to reconstruct the model, which is then applied to predict cone crusher liner wear. The crushing force P exerted by the liner on the material can be decomposed into the normal force $P_{n}$ perpendicular to the liner surface and the tangential force $P_{s}$, expressed as:

(12) $$P_{n}=\frac{P}{{\sqrt{1+(\tan\frac{\alpha }{2})}}^{2}}$$

(13) $$P_{s}=\frac{P\tan\frac{\alpha }{2}}{\sqrt{1+{\left(\tan\frac{\alpha }{2}\right)}^{2}}}$$

(14) $$\Delta \omega =\frac{P_{n}+KP_{s}}{W_{r}}$$

Considering that during the operation of a cone crusher, the contact time between the ore and the mantle liner is significantly longer than that with the concave liner, and the sliding and compression actions on the mantle are more complex, the wear relationship for the concave proposed by Lindqvist et al. is adopted as the base model in the modeling process. This model is then modified for the calculation of mantle wear. Through a comprehensive analysis of the force characteristics and contact behavior of the mantle and concave, a transformation relationship between their wear amounts is established. This relationship can be used to map the wear profile of the concave to that of the mantle, enabling dynamic prediction of wear distribution.

(15) $$\Delta \omega_{D}=\frac{p_{\max}}{W_{r}}$$

By combining Equations (12)–(15), the transformation formula for the wear amount between the moving cone and the fixed cone is obtained as:

(16) $$\Delta \omega_{d}=\frac{\Delta \omega_{D}(1+K\cdot \tan\frac{\alpha }{2})}{\sqrt{1+{\left(\tan\frac{\alpha }{2}\right)}^{2}}}$$

where $\Delta \omega_{d}$ represents the wear amount of the moving cone.

**Figure 18:**
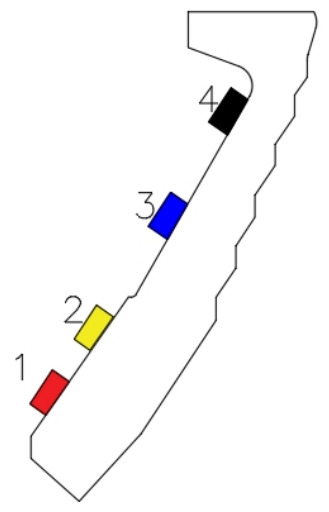
The position of the sensor on the fixed cone liner.

To validate the model’s rationality, ultrasonic sensors were used to monitor the wear of the fixed cone liner in an actual piece of equipment. The sensors were evenly arranged at different heights on the surface of the fixed cone liner, with their installation positions shown in Figure 18. By collecting data during operation, the variations in liner thickness at each monitoring point under different operating times were obtained.

**Figure 19:**
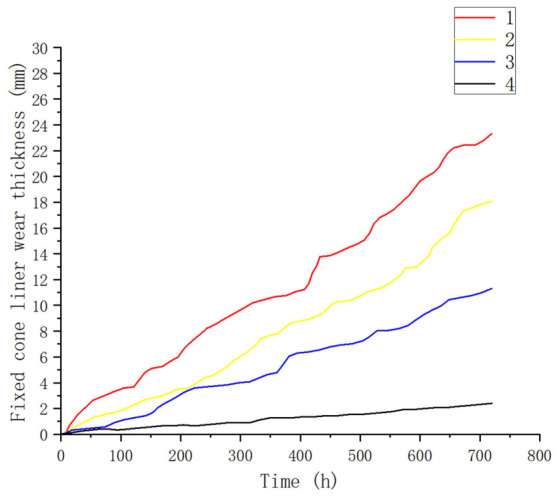
Wear thickness curve graph of the fixed cone liner.

The liner wear curve is shown in Figure 19. It can be observed that the wear amount of the liner increases cumulatively with the operating time of the equipment, and the wear in the lower part of the crushing chamber is significantly more severe than that in the upper part. This is mainly attributed to the longer residence time of materials in this region, as well as the more intense compression and impact forces they experience.

**Figure 20:**
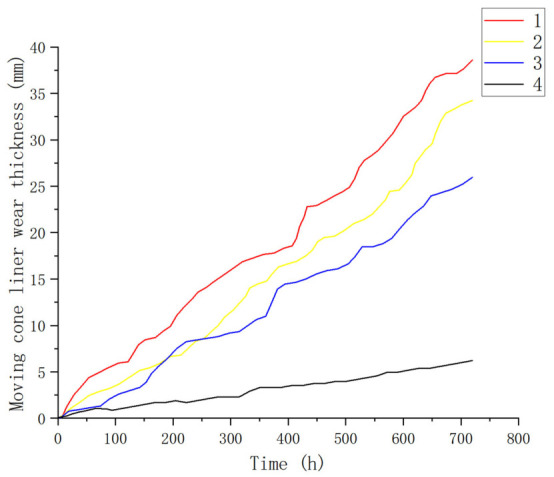
Wear thickness curve graph of the moving cone liner.

The calculation parameters for the physical model were obtained from the dimensional design drawing of the liner, and the sample wear-point data were transformed using the model. The physical model was primarily calculated based on linear parameters. The wear thickness curve of the moving cone liner, calculated using Equation (16), is shown in Figure 20.

**Figure 21:**
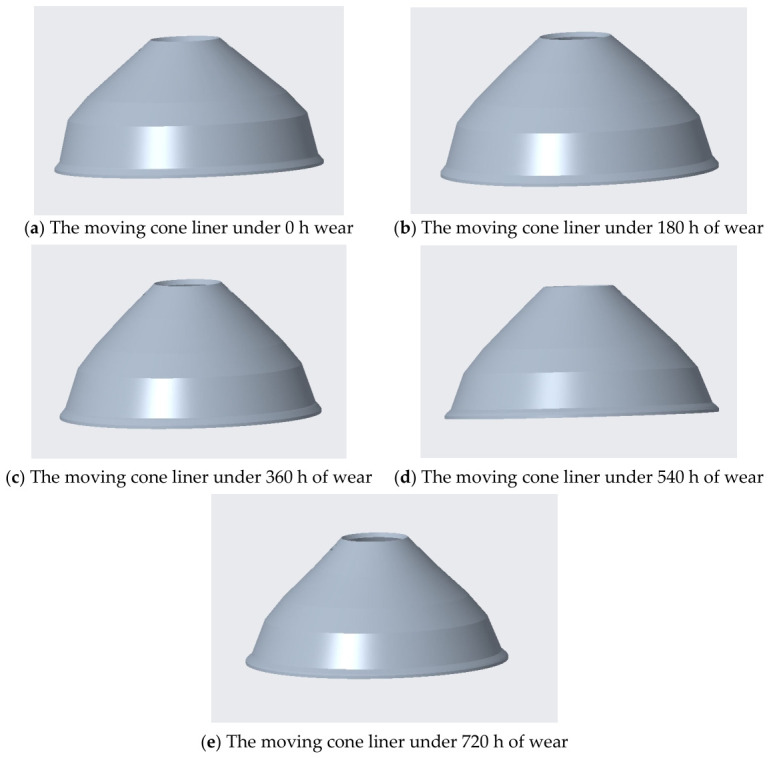
Three-dimensional models of different worn moving cone liners.

Based on the analysis of the wear mechanism [22], a calculation model for the wear amount of the moving cone liner was established and validated using ultrasonic measurement data. Five wear stages—0 h, 180 h, 360 h, 540 h, and 720 h—were selected, and corresponding three-dimensional models were created in Creo, as shown in Figure 21.

**Figure 22:**
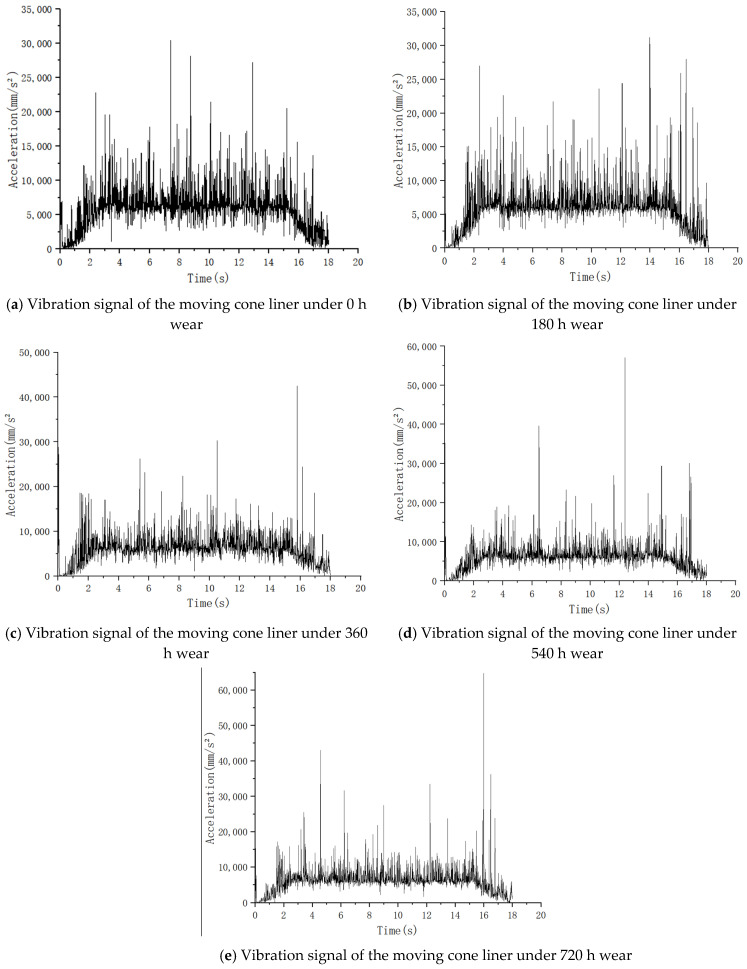
Vibration signals of the moving cone liner at different time periods.

From the figure, it is clearly observed that with the accumulation of operating time, the surface material of the liner gradually peels off, leading to significant changes in its contour morphology. The wear area expands from local to the entire surface, with the lower part being particularly affected. To quantitatively analyze the influence of deepening wear on the vibration response of the moving cone, the liner models under different wear states were sequentially replaced into the parameter-optimized virtual prototype of the cone crusher in ADAMS (with the optimized counterweight of 5100 N and rotational speed of 381 rpm maintained). Dynamic simulations were conducted, and the acceleration vibration signals at the centroid of the moving cone liner were extracted. Figure 22 presents the typical acceleration time-domain waveforms during steady-state operation at five wear stages. This method follows the “measurement-modeling-simulation” workflow: first, the wear thickness distribution of the moving cone liner at each stage is obtained based on the measured moving cone wear data and the moving-fixed cone transformation relationship. Subsequently, the corresponding wear models are reconstructed in Creo and imported into the ADAMS virtual prototype. Acceleration vibration signals are extracted under unified working conditions, enabling the systematic reproduction of vibration responses under different wear degrees without interrupting equipment operation, thus laying a foundation for subsequent health state prediction.

By comparing the signals in Figure 22, it can be observed that as wear intensifies (from a to e), the vibration signal amplitude generally increases and the waveforms become more complex. High-frequency components and impact characteristics become increasingly pronounced, which intuitively reflects the influence of liner wear on the system’s dynamic characteristics.

### 4.2. Health Feature Extraction and Dimensionality Reduction

To quantitatively characterize the variation in vibration signals associated with wear degradation, twelve time-domain and frequency-domain characteristic parameters were extracted from the vibration signals at each stage, forming the initial health feature set. The selected features refer to the widely used indicator system in the field of mechanical health monitoring [23]. Among the time-domain features, dimensional indicators (e.g., mean, variance) reflect vibration energy and structural stiffness variations. The time-domain features include *F*1 (mean), *F*2 (mean square value), *F*3 (variance), and *F*4 (standard deviation). Dimensionless indicators (e.g., kurtosis, skewness) are more sensitive to late-stage impacts and unstable vibrations, and include *F*5 (kurtosis), *F*6 (kurtosis factor), *F*7 (skewness), and *F*8 (skewness factor). Frequency-domain features (e.g., dominant frequency, spectral energy) reveal the evolution of wear patterns from the perspectives of energy distribution and frequency shift. The frequency-domain features (obtained through Fast Fourier Transform of the signals) include *F*9 (dominant frequency), *F*10 (mean square frequency), *F*11 (spectral centroid), and *F*12 (spectral energy). The above features account for the characterization requirements of different degradation stages, providing a basis for subsequent dimensionality reduction and health indicator construction. The calculation formulas for commonly used time-domain characteristic parameters are shown in Table 3, while the calculation formulas for key indicators reflecting the vibration intensity and stress state of the moving cone liner are presented in Table 4.

Since features were extracted from signals at only five discrete time points, the data points are sparse. To obtain a continuous feature evolution trend, Lagrange interpolation was first applied to the feature values at these five time points, followed by smoothing fitting using a quadratic polynomial to derive continuously varying characteristic curves from 0 to 720 h. Subsequently, to address potential redundancy and correlation among the 12 features, Principal Component Analysis (PCA) was employed to reduce dimensionality [24]. The procedure is described as follows.

The feature data of the moving cone liner at different wear stages (0 h, 180 h, 360 h, 540 h, and 720 h) form a feature matrix.

(17) $$X={\left[x_{ij}\right]}_{5\times 12}$$

In the equation, $x_{ij}$ represents the j-th feature value at the i-th time instant.

To obtain continuous, smoothly evolving temporal characteristics, Lagrange interpolation and smoothing quadratic polynomial fitting were applied to each feature sequence $\left\{\left(t_{i},x_{ij}\right)\right\}$.

Let the sampling time instants be: $t_{1}=0,t_{2}=180,t_{3}=360,t_{4}=540,t_{5}=720\left(h\right)$

The corresponding feature values are $y_{i}^{\left(j\right)}=x_{ij}$.

The Lagrange interpolation polynomial is defined as:

(18) $$f_{j}^{\left(lag\right)}\left(t\right)=\sum_{i=1}^{5}y_{i}^{\left(j\right)}L_{i}\left(t\right)$$

Among them

(19) $$L_{i}\left(t\right)=\prod_{\begin{array}{l} m=1\\ m\neq i \end{array}}^{5}\frac{t-t_{m}}{t_{i}-t_{m}}$$

where $L_{i}(t)$ denotes the i-th Lagrange basis function.

To address interpolation errors and experimental noise, a quadratic polynomial smoothing is applied to the interpolation results.

Let the fitting function be expressed as:

(20) $$p_{j}\left(t\right)=a_{0}^{\left(j\right)}+a_{1}^{\left(j\right)}t+a_{2}^{\left(j\right)}t^{2}$$

where $a_{0}^{\left(j\right)}$, $a_{1}^{\left(j\right)}$, and $a_{2}^{\left(j\right)}$ are the constant, linear, and quadratic coefficients, respectively.

The least squares criterion is given by:

(21) $$\underset{a_{0}^{j},a_{1}^{j},a_{2}^{j}}{\min}{\sum_{q=1}^{N}\left(f_{j}^{\left(lag\right)}\left(t_{q}\right)-p_{j}\left(t_{q}\right)\right)}^{2}$$

The parameter vector is obtained via the normal equation:

(22) $$a^{\left(j\right)}=\left[\begin{array}{l} a_{0}^{\left(j\right)}\\ a_{1}^{\left(j\right)}\\ a_{2}^{\left(j\right)} \end{array}\right]={\left(V^{T}V\right)}^{-1}V^{T}y^{\left(j\right)}$$

where $V={\left[\begin{array}{ccc} 1 & t_{q} & t_{q}^{2} \end{array}\right]}_{N\times 3}$ denotes the Vandermonde matrix, and

$$y^{\left(j\right)}={\left[f_{j}^{\left(lag\right)}\left(t_{1}\right),\cdots ,f_{j}^{\left(lag\right)}\left(t_{N}\right)\right]}^{T}$$

After fitting, the smoothed characteristic curve is obtained as $f_{j}\left(t\right)=p_{j}\left(t\right)$.

**Figure 23:**
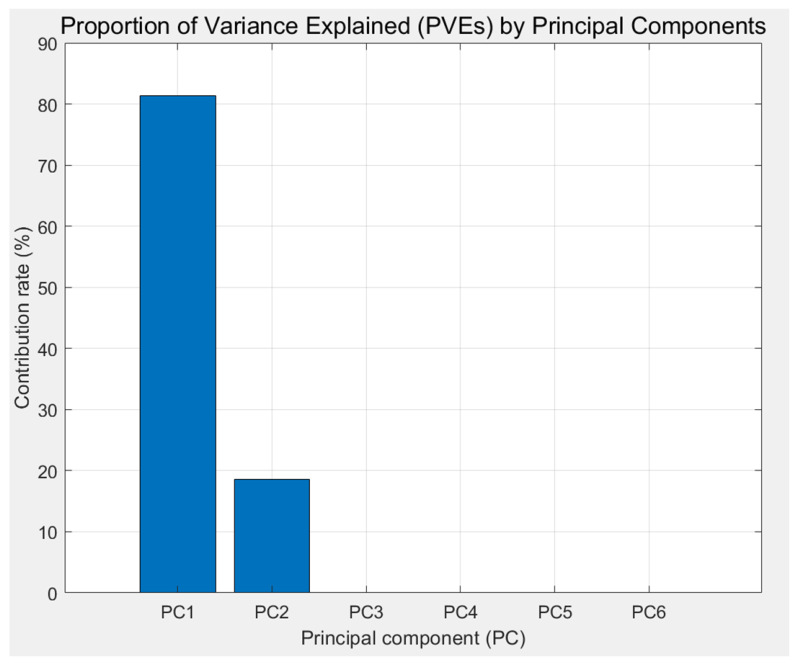
Principal component contribution rate.

The smoothed feature matrix $F=\left[f_{1}\left(t\right),\cdots ,f_{12}\left(t\right)\right]$ is normalized and then used as input to the PCA model. The resulting contribution rates of the principal components are shown in Figure 23.

As shown in Figure 23, the first principal component (PC1) accounts for 77% of the total variance, while the second principal component (PC2) contributes 22%. Together, the cumulative contribution rate of the first two principal components reaches 99%, indicating that they capture nearly all the information embedded in the original 12 features. Analysis of the principal component loading matrix reveals that PC1 is predominantly driven by features *F*1, *F*2, *F*3, *F*4, and *F*12, which are strongly associated with the overall energy and intensity of the signal. Accordingly, PC1 can be characterized as an “energy-related” principal component. In contrast, PC2 is mainly driven by features *F*5, *F*6, *F*10, and *F*11, which are closely related to the signal’s impulsiveness and frequency distribution. Thus, PC2 can be interpreted as an “impulsiveness-related” principal component. Consequently, PC1 and PC2 are selected as the core inputs for constructing subsequent health indicators. This approach not only achieves dimensionality reduction but also retains the essential information reflective of the degradation process.

### 4.3. Adaptive Feature Fusion and Health Indicator Construction

To further improve the sensitivity and robustness of health degree characterization, an Adaptive Spatial Feature Fusion (ASFF) model is introduced. By dynamically weighting multi-source health factors at the feature level, it realizes adaptive optimal fusion of different features under time-varying environments. In this paper, ASFF is selected for feature fusion mainly due to its adaptive weight adjustment capability. The vibration signals of cone crushers exhibit strong time-varying and non-stationary characteristics; the contribution degrees of energy-type features and impact-type features dynamically change across different degradation stages, making traditional fixed-weight fusion strategies difficult to adapt to such working conditions. In a multi-scale feature fusion study, He Mingjie et al. systematically compared the performance of ASFF with various attention mechanisms including SE, CBAM, ECA, GAM, and Coord, and the results showed that ASFF significantly outperformed these methods in improving model performance [25]. Additionally, Zhan et al. [26] applied attention mechanism-based adaptive feature fusion to bearing performance evaluation, proving that compared with the PCA fixed-weight method, the consistency of health indicators can be improved by 18.7%, which further supports the method selection in this paper. The model inputs include multiple principal component features from the PCA stage and their extended health factor set:

(23) $$X\left(t\right)=\left\{H_{1}\left(t\right),H_{2}\left(t\right),\cdots ,H_{m}\left(t\right)\right\}$$

where $H_{m}\left(t\right)$ represents the health features from different sources.

The output of the model is the comprehensive health indicator:

(24) $$\hat{H}\left(t\right)=ASFF\left(X\left(t\right)\right)$$

which denotes the adaptively fused health degree at time t.

ASFF computes weights for each input feature channel at every time step. Let $\omega_{m}(t)$ denote the weight assigned to the m-th feature. The fusion formulation is then given by:

(25) $$\hat{H}\left(t\right)=\sum_{m=1}^{M}\omega_{m}\left(t\right)H_{m}\left(t\right)$$

(26) $$\sum_{m=1}^{M}\omega_{m}\left(t\right)=1$$

where M denotes the number of feature channels;

$H_{m}(t)$ represents the m-th health feature;

$\omega_{m}(t)$ is the adaptive weight generated by the ASFF module, which takes the form:

(27) $$\omega_{m}\left(t\right)=\frac{\exp\left(f_{m}\left(X\left(t\right)\right)\right)}{\sum_{j=1}^{M}\exp\left(f_{j}\left(X\left(t\right)\right)\right)}$$

where $f_{m}(\cdot )$ is a nonlinear mapping constructed by fully connected or convolutional layers, used to evaluate the contribution of each feature to the overall health indicator.

The ASFF module aims to minimize the deviation between the predicted health indicator and the true health labels, and is optimized using the mean squared error (MSE) loss function:

(28) $$\varsigma =\frac{1}{N}{\sum_{i=1}^{N}\left(\hat{H}\left(t_{i}\right)-H_{ref}\left(t_{i}\right)\right)}^{2}$$

where $H_{\mathrm{ref}}(t_{i})$ denotes the reference health indicator obtained from experiments or simulations, and $\hat{H}(t_{i})$ is the health indicator predicted by the model.

**Figure 24:**
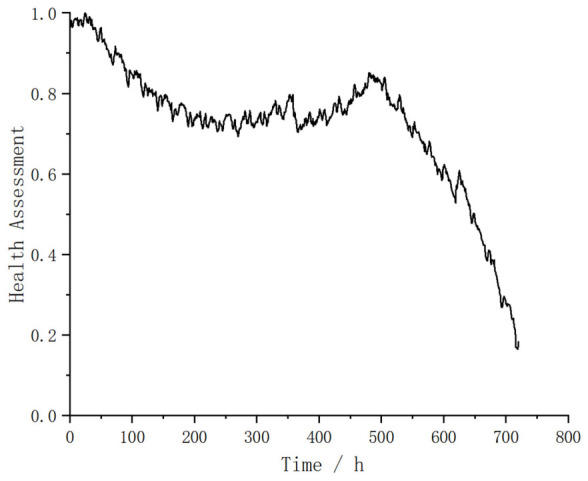
The health curve generated by the ASFF model.

The health indicator curve generated by the ASFF model after training is shown in Figure 24.

As shown in the figure, the curve clearly presents the three-stage degradation process of the moving cone liner: the health degree rapidly decreases from 1 to approximately 0.80 within 0–150 h, corresponding to the initial running-in period; it then fluctuates slightly between 0.75 and 0.80 during 150–450 h, representing the stable wear period; a slight rise in health degree occurs from 450 to 550 h, forming a mid-term fluctuating recovery stage; finally, the decline rate accelerates significantly after 550 h, entering the accelerated degradation stage. The curve is generally smooth with good monotonicity, providing a reliable data basis for subsequent life extrapolation and state division.

### 4.4. Weibull Degradation Prediction and Life Assessment

After obtaining the smoothed health indicator curve H(t), the Weibull degradation model is used to fit and extrapolate the curve for life prediction [27,28]. The applied model is given by:

(29) $$H\left(t\right)=\exp\left[-{\left(\frac{t}{\eta }\right)}^{\beta }\right]$$

where β is the shape parameter;

η is the scale parameter.

Taking the logarithmic transformation of the health indicator yields a linear form:

(30) $$\ln\left[-\ln H\left(t\right)\right]=\beta \ln t-\beta \ln\eta$$

The parameters β and η are then estimated by fitting ln[−lnH(t)] against lnt using least squares regression.

The Weibull model was then extrapolated to t=900 h to obtain the degradation trend of the health indicator during the prediction phase.

**Figure 25:**
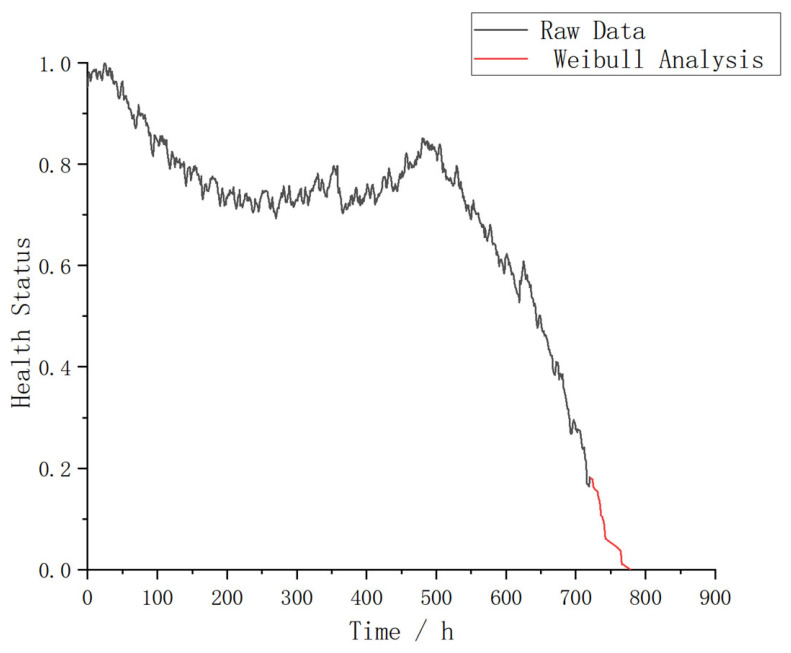
The health curve of Weibull fitting and extrapolation prediction.

Figure 25 presents the polynomial-fitting result for the ASFF-based comprehensive health indicator curve, along with its extrapolated prediction trend based on the Weibull model.

As shown in Figure 25, the overall health indicator curve exhibits a three-stage degradation pattern. The Weibull curve provides a good fit to the accelerated degradation phase in the later stages, and the predicted trend aligns well with the original data. In the early stages of operation, the health indicator drops rapidly from 1 to approximately 0.80 within the first 150 h, corresponding to the running-in of the contact surfaces, where wear is relatively rapid but gradually stabilizes. Subsequently, the curve enters a steady and slowly varying stage, during which the health indicator fluctuates slightly between 0.75 and 0.80, reflecting the steady-state wear period of the moving cone liner. Between 450 and 550 h, a slight rebound in the health indicator is observed, forming a mid-term fluctuation and recovery phase, which may be attributed to reduced material impact or the smoothing effect of the algorithm. From 550 h onward, the curve enters an accelerated degradation stage, where the health indicator declines sharply from approximately 0.8 to 0.15–0.20. This phase corresponds to severe wear in the liner’s later service life and represents a critical period approaching failure, requiring focused monitoring.

The Weibull model assumes accelerated degradation; therefore, the predicted curve is smooth and monotonically decreasing, effectively eliminating stochastic noise in the original signal while preserving the sharp decline in the later stages.

The predicted segment aligns well with the steep degradation trend at the end of the original black curve, indicating that the Weibull model captures the dominant degradation trend and that the fitting results are credible. Extrapolation using the Weibull model shows that the health indicator approaches zero at approximately 785 h, suggesting that the liner may reach its wear limit or failure threshold around this time. Consequently, it is recommended to schedule maintenance or replacement before this point.

### 4.5. Health State Classification and Evaluation of the Moving Cone Liner

#### 4.5.1. Application of FCM Clustering Algorithm in Health State Division

The health indicator data within the time interval are denoted as:

(31) $$H=\left\{H\left(t_{1}\right),H\left(t_{2}\right)\cdots ,H\left(t_{N}\right)\right\},H\left(t\right)\in \left(0,1\right)$$

According to the health management requirements of the equipment, the health state is categorized into four classes:

c=4

representing four typical operating states: healthy, good, degraded, and faulty, respectively.

Based on the following formulation:

(32) $$J_{m}(U,V)=\sum_{i=1}^{n}\sum_{j=1}^{c}u_{ij}^{m}{\left\|H\left(t_{i}\right)-v_{j}\right\|}^{2}$$

The solution is obtained iteratively between the cluster centers $v_{j}$ and the membership matrix $U=[u_{ij}]$. The fuzzy index is set to m=2 to maintain moderate sensitivity to boundary samples.

**Figure 26:**
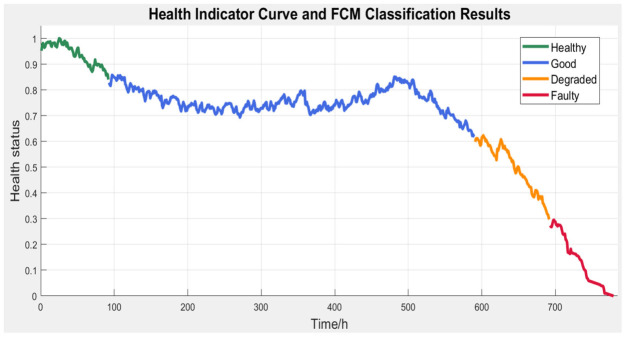
FCM classification results of health status curves.

Continuous health indicator values are used to interpret health states using the Fuzzy C-Means (FCM) clustering algorithm. With the number of clusters set to C=4, the continuous H(t) data points are classified into four state categories: “healthy,” “good,” “degraded,” and “faulty,” according to the maximum membership degree principle. The resulting classification is shown in Figure 26, where the four state intervals are clearly distinguished by different background colors.

#### 4.5.2. Analysis of Clustering Results for Health States at Different Stages

To further clarify the operating stages and health-level characteristics corresponding to each health state, a statistical analysis was conducted on the time intervals and health indicator distributions for each state, based on the FCM clustering results. The findings are presented in Table 5.

From the analysis in Table 5, the health status is classified into four categories. This classification is primarily based on the following: from the perspective of physical mechanisms, the wear of the dynamic liner follows a three-stage degradation pattern of “initial running-in → steady-state wear → rapid failure”; from the perspective of engineering practice, a four-level early warning system is commonly adopted in industrial equipment maintenance. The four-category classification takes into account both the degradation mechanism and operational requirements. Each threshold is closely related to the wear mechanism: 0.84 corresponds to the end of the running-in period, 0.62 marks the onset of accelerated degradation, and 0.30 indicates the critical point of severe failure—all of which have clear physical significance.

**Figure 27:**
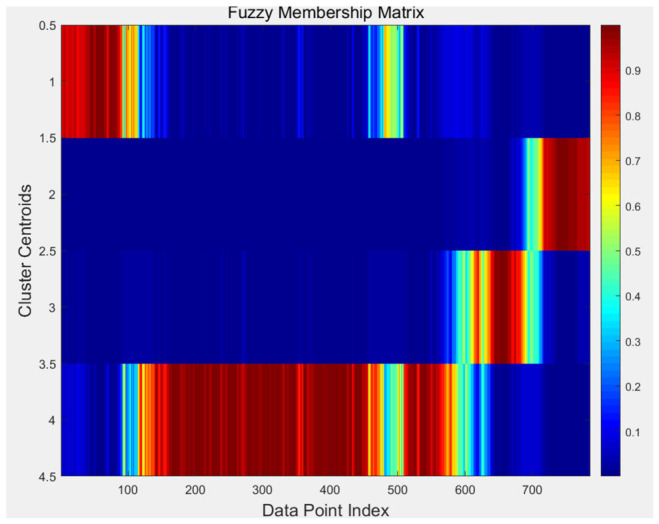
Membership degree matrix.

To validate the effectiveness of the clustering and the clarity of sample assignment, a visualization of the membership matrix is presented in Figure 27.

As shown in the analysis of Figure 27, the system’s health state evolves and can be divided into four distinct stages. The first stage, from 0 to 92 h, corresponds to the healthy state, where the membership degree is close to 1, clearly placing the samples in the “healthy” category with well-defined cluster boundaries. The second stage, from 93 to 590 h, represents the good state, during which the membership degrees exhibit a banded distribution and gradually transition; although the health indicator fluctuates, it remains within the “good” level. The third stage, from 591 to 692 h, marks the degradation state, characterized by a sudden increase in membership degrees as samples rapidly converge toward the degradation cluster, accompanied by a significant decline in the health indicator, indicative of accelerated wear. Finally, the fourth stage, from 693 to 785 h, corresponds to the faulty state, where the membership degrees concentrate near 1, indicating that the system health has reached a failure condition. This result is consistent with the end-of-life point predicted by the Weibull extrapolation.

**Figure 28:**
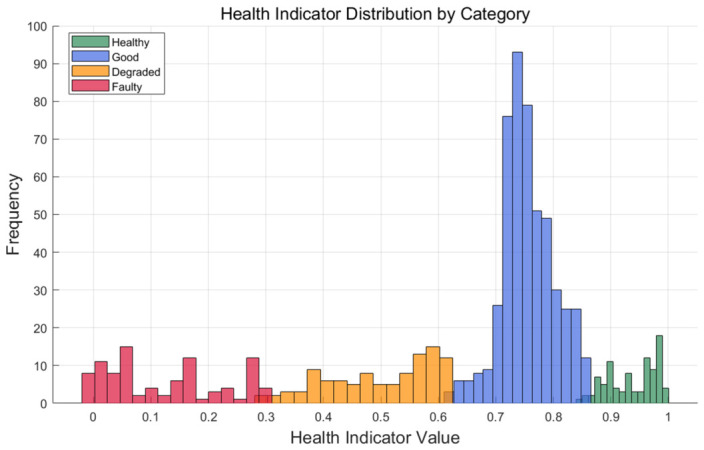
Health degree distribution.

To verify the reasonableness of the health indicator thresholds determined by the FCM clustering boundaries, distribution histograms of the health indicator values are plotted for each state category, as shown in Figure 28. It can be observed that the distributions of health indicator values within each category are relatively concentrated, with clear boundaries between categories, which demonstrates the validity of the clustering division.

As shown in Figure 28, the healthy samples are highly concentrated in the 0.84–1 range, with a distinct peak, indicating slight wear in the early stage and stable vibration characteristics. The good samples display the widest distribution, mainly ranging from 0.62 to 0.84, corresponding to the typical steady wear stage. The degraded samples are concentrated in the lower-middle range, primarily between 0.30 and 0.62, with a broader distribution reflecting reduced structural stiffness and increased impact components. The faulty samples are concentrated in the 0–0.30 range, representing the end-of-life stage characterized by intense vibration impacts and significant randomness.

The distribution plots are consistent with the trend observed in the membership matrix, further demonstrating the statistical reasonableness of the health state interval divisions presented in Table 5.

The FCM clustering algorithm not only automatically segments the health indicator curve but also accurately reveals the complete degradation mechanism of the moving cone liner from a healthy state to failure. The four health states exhibit clear time intervals and well-defined health indicator distributions, which are highly consistent with the original health indicator trend and the Weibull prediction results. This provides a reliable basis for subsequent equipment condition monitoring, health threshold setting, and maintenance strategy development.

## 5. Discussion

The comprehensive framework developed in this study, integrating dynamic simulation, intelligent optimization, and data-driven methods, has achieved significant results in both parameter optimization and health prediction for cone crushers’ moving cone liners.

At the algorithmic level, the Improved Butterfly Optimization Algorithm (IBOA) significantly enhances global search capability and convergence accuracy through Cauchy mutation and adaptive weighting, outperforming the standard BOA on multiple benchmark functions. Applied to the collaborative optimization of counterweight and rotational speed, the obtained optimal parameter combination (5100 N, 381 rpm) markedly improves the smoothness of system operation. The optimization mechanism lies in the following: the increased counterweight offsets the inertial centrifugal force, while the higher rotational speed better matches the crushing frequency with the laminating frequency, thereby improving energy efficiency. From an engineering perspective, the 15.4% reduction in the standard deviation of force directly lowers the fatigue stress amplitude of key transmission components, contributing to extended trouble-free operation time. Meanwhile, the simultaneous decrease in both the coefficient of variation and the crest factor indicates that load fluctuations and impact components are effectively suppressed. The average force increases by only 0.9% after optimization, suggesting that the improvement in operational smoothness is achieved while maintaining crushing capacity, aligning with the engineering goal of “maintaining throughput while enhancing stability.” Compared with studies focusing only on static structural parameters, the collaborative optimization of dynamic operating parameters in this paper is more relevant to actual working conditions.

In terms of health state prediction, the proposed combined model—comprising “PCA dimensionality reduction + ASFF fusion + Weibull extrapolation + FCM clustering”—enables continuous quantification and automatic classification of the liner degradation process. After PCA dimensionality reduction of the time- and frequency-domain features extracted from vibration signals, the first two principal components reflect energy intensity and impact characteristics, respectively, which are consistent with the wear mechanism [22]. The health indicator curve generated by adaptive ASFF fusion exhibits a typical three-stage degradation pattern. The Weibull model extrapolation predicts that the health indicator approaches zero at 785 h, aligning well with the measured wear trend [28]. Based on FCM clustering, the health indicator is automatically classified into four stages: healthy, good, degraded, and faulty. The cluster centers of the four stages are 0.92, 0.76, 0.48, and 0.15, respectively, showing a clear decreasing trend. The corresponding interval lengths are 92 h, 497 h, 101 h, and 92 h, with well-defined sample boundaries and statistically reasonable clustering results. This classification provides a quantitative basis for equipment maintenance: normal operation in the healthy stage, enhanced monitoring in the good stage, scheduled maintenance in the degraded stage, and immediate shutdown in the faulty stage. Compared with traditional threshold-based alarming, this method achieves early warning and refined classification of progressive degradation, offering greater engineering practicality [16].

At the same time, it must be acknowledged that this study has certain limitations. The simulation assumes homogeneous granite as the material, which deviates from the variable properties of actual ores. Due to simulation costs, the degradation curve is interpolated based on only five wear stages, which may affect the local objectivity of the degradation curve. The Weibull model has limited capability in characterizing non-monotonic degradation. The number of FCM clusters needs to be adjusted according to the actual equipment condition. Future research will increase the sampling density to quantify the interpolation uncertainty. In terms of method generalizability, the proposed optimization and health assessment framework can be transferred to other wear-prone components in mining machinery. By updating the material parameters and the crushing force model, the approach can be extended to different ore types and equipment models. Compared with existing methods, the advantage of this paper lies in integrating parameter optimization and health prediction within a single framework. The ASFF-fused indicator is more robust, and the FCM-based classification enables quantitative early warning. In engineering implementation, the response surface of the optimized parameters can serve as an offline reference model, combined with closed-loop speed control to improve operational smoothness. Meanwhile, online vibration monitoring and real-time feature fusion can generate the health indicator, enabling predictive maintenance based on the four-level thresholds. The proposed method is thus feasible for transplantation into industrial control systems. Future research can further integrate online monitoring and digital twin technology, extend the approach to multi-component coupled modeling, and introduce uncertainty quantification to enhance prediction accuracy.

## 6. Conclusions

This study presents a comprehensive investigation into performance enhancement and intelligent operation and maintenance requirements for critical components of cone crushers, integrating dynamic analysis, parameter optimization, and health-state prediction. The main conclusions are as follows:

(1) The Improved Butterfly Optimization Algorithm (IBOA) significantly enhances global search capability and convergence accuracy through Cauchy mutation and adaptive weight strategies. After the collaborative optimization of counterweight and rotational speed, the standard deviation of the mantle’s force is reduced by 15.4%, and the coefficient of variation decreases by 16.3%, resulting in a substantial improvement in system operation stability.

(2) The proposed integrated model, consisting of “PCA dimensionality reduction + ASFF fusion + Weibull extrapolation + FCM clustering”, achieves continuous quantification and automatic classification of the liner degradation process. The health degree curve exhibits a three-stage degradation pattern: initial running-in → stable wear → accelerated failure. Weibull extrapolation predicts that the liner will enter the fault risk period at approximately 785 h.

(3) FCM clustering automatically divides the health status into four stages: healthy, good, degraded, and faulty. The corresponding health degree thresholds for each stage are 0.84, 0.62, and 0.30, respectively. The interval boundaries are clear and highly consistent with the degradation trend, providing a reliable basis for equipment condition monitoring and graded maintenance.

## Figures and Tables

**Table 1:** Setting of contact friction force parameters.

Component	Normal Force (N)	Stiffness (N/mm)	Force Exponent	Damping (N·s/mm)	Penetration Depth (mm)	Static Friction Coefficient	Dynamic Friction Coefficient
Drive Gear & Driven Gear	Impact	1.0 × 10^+5^	1.6	30	0.1	9.0 × 10^−2^	6.0 × 10^−2^
Eccentric Sleeve & Moving Cone	Impact	1.0 × 10^+5^	1.6	50	0.1	9.0 × 10^−2^	6.0 × 10^−2^

**Table 2:** Optimize the comparison of stress indexes of the front and rear moving cone liners.

Parameter	Before Optimization	After Optimization	Rate of Change
Mean (N)	1.156 × 10^5^	1.166 × 10^5^	0.9%
Standard deviation (N)	1.49 × 10^3^	1.26 × 10^3^	−15.4%
Coefficient of variation (%)	1.29	1.08	−16.3%
Peak-to-peak value (N)	1.31 × 10^4^	1.25 × 10^4^	−4.6%
Peak factor	1.045	1.036	−0.86%

**Table 3:** Mathematical expression of time-domain characteristic parameters.

Feature Parameter	Calculation Formula	Feature Parameter	Calculation Formula
Mean	$X_{\mu }=\frac{1}{N}\sum_{i=1}^{N}x_{i}$	Mean square value	$X_{ms}=\frac{1}{N}\sum_{i=1}^{N}x_{i}^{2}$
Variance	$X_{\sigma^{2}}=\frac{1}{N}\sum_{i=1}^{N}{\left(x_{i}-\mu \right)}^{2}$	Standard deviation	$X_{\sigma }=\sqrt{\frac{1}{N}\sum_{i=1}^{N}{\left(x_{i}-\mu \right)}^{2}}$
Kurtosis	$X_{K}=\frac{\frac{1}{N}\sum_{i=1}^{N}{\left(x_{i}-\mu \right)}^{4}}{{\left(\frac{1}{N}\sum_{i=1}^{N}{\left(x_{i}-\mu \right)}^{2}\right)}^{2}}$	Kurtosis factor	$X_{Kf}=\frac{X_{K}}{3}$
Skewness	$X_{\gamma }=\frac{\frac{1}{N}\sum_{i=1}^{N}{\left(x_{i}-\mu \right)}^{3}}{{\left(\sqrt{\frac{1}{N}\sum_{i=1}^{N}{\left(x_{i}-\mu \right)}^{2}}\right)}^{3}}$	Skewness factor	$X_{\gamma f}=\left|\frac{\frac{1}{N}\sum_{i=1}^{N}{\left(x_{i}-\mu \right)}^{3}}{{\left(\sqrt{\frac{1}{N}\sum_{i=1}^{N}{\left(x_{i}-\mu \right)}^{2}}\right)}^{3}}\right|$

**Table 4:** Mathematical expression of frequency-domain characteristic parameters.

Feature Parameter	Formula	Feature Parameter	Formula
Dominant frequency	$X_{f\max}=\arg\max\left(P\left(f\right)\right)$	Spectral centroid	$X_{fms}=\frac{\sum_{i=1}^{N}f_{i}^{2}P_{i}}{\sum_{i=1}^{N}P_{i}}$
Mean square frequency	$X_{fc}=\frac{\sum_{i=1}^{N}f_{i}P_{i}}{\sum_{i=1}^{N}P_{i}}$	Spectral energy	$X_{E_{s}}=\sum_{i=1}^{N}P_{i}^{2}$

**Table 5:** Health status intervals and health degree distribution.

Status	Start Time (h)	End Time (h)	Min Health Indicator	Max Health Indicator
Healthy	0	92	0.84	1.00
Good	93	590	0.62	0.84
Degraded	591	692	0.30	0.62
Faulty	693	785	0	0.30

Note: The four states are derived using the Fuzzy C-Means (FCM) clustering algorithm with a fuzzy index m = 2. The health degree thresholds are set as follows: 0.84 (between healthy and good), 0.62 (between good and degraded), and 0.30 (between degraded and faulty). The start and end times correspond to the intervals of each state.

## Data Availability

The raw data supporting the conclusions of this article will be made available by the authors upon request.
